# Preferential Binding of Mg^2+^ Over Ca^2+^ to CIB2 Triggers an Allosteric Switch Impaired in Usher Syndrome Type 1J

**DOI:** 10.3389/fnmol.2018.00274

**Published:** 2018-08-17

**Authors:** Rosario Vallone, Giuditta Dal Cortivo, Mariapina D'Onofrio, Daniele Dell'Orco

**Affiliations:** ^1^Section of Biological Chemistry, Department of Neurosciences, Biomedicine and Movement Sciences, University of Verona, Verona, Italy; ^2^Department of Biotechnology, University of Verona, Verona Italy

**Keywords:** calcium sensor, Usher syndrome 1J, DFNB48, nuclear magnetic resonance, hearing loss, calcium and integrin binding protein, allostery, magnesium

## Abstract

Calcium and integrin binding protein 2 (CIB2) shares with the other members of the CIB family the ability to bind Ca^2+^ and Mg^2+^ via two functional EF-hand motifs, namely EF3 and EF4. As a cation sensor, CIB2 is able to switch to a conformation likely associated with specific biological functions yet to be clarified. Recent findings demonstrate the involvement of CIB2 in hearing physiology and a single, conservative point mutation (p.E64D) has been related to Usher Syndrome type 1J (USH1J) and non-syndromic hearing loss. We present an exhaustive biochemical and biophysical characterization of human wild type (WT) and E64D CIB2. We found that CIB2 does not possibly work as a calcium sensor under physiological conditions, its affinity for Ca^2+^ (K_d_^app^ = 0.5 mM) being too low for detecting normal intracellular levels. Instead, CIB2 displays a significantly high affinity for Mg^2+^ (K_d_^app^ = 290 μM), and it is probably Mg^2+^ -bound under physiological conditions. At odds with the homologous protein CIB1, CIB2 forms a non-covalent dimer under conditions that mimic the physiological ones, and as such it interacts with its physiological target α7B integrin. NMR spectroscopy revealed a long-range allosteric communication between the residue E64, located at the N-terminal domain, and the metal cation binding site EF3, located at the C-terminal domain. The conservative E64D mutation breaks up such inter-domain communication resulting in the impaired ability of CIB2 to switch to its Mg^2+^-bound form. The ability to bind the target integrin peptide was substantially conserved for E64D CIB2, thus suggesting that the molecular defect associated with USH1J resides in its inability to sense Mg^2+^ and adopt the required conformation.

## Introduction

Calcium and integrin binding protein 2 (CIB2) is a 21.6 kDa protein sharing with the other members of the CIB family the ability to bind Ca^2+^ and Mg^2+^ via two functional EF-hand motifs, namely EF3 and EF4, therefore switching to a specific conformation likely associated with specific biological functions (Huang et al., 2012). Since its discovery as a ubiquitously expressed DNA-dependent protein kinase interacting protein (Seki et al., 1999), CIB2 has been found to be expressed in a variety of tissues but its physiological role remains largely unknown. It has been established that CIB2 binds specifically to the integrin α7B cytoplasmic domain (Häger et al., 2008; Huang et al., 2012), however the protein interacts also with the αIIb integrin (Huang et al., 2012), thus broadening its potential involvement in a variety of signal transduction processes.

Recent lines of evidence demonstrate the direct involvement of CIB2 in hearing physiology, as CIB2 knockout mice showed abolished mechanoelectrical transduction in auditory cells leading to profound hearing loss (Wang et al., 2017). Interestingly, four missense mutations in the gene encoding for CIB2 [p.F91S, p.C99W, p.I123T (Riazuddin et al., 2012) and more recently p. R186W (Patel et al., 2015)] have been found to be associated with non-syndromic deafness (DFNB48) while a single, conservative point mutation (p.E64D) (Riazuddin et al., 2012) has been related to Usher Syndrome type 1J (USH1J, OMIM entry: 614869), a genetic disorder characterized by hearing loss and progressive vision loss due to retinitis pigmentosa. Altogether, these recent findings suggest that CIB2 is an essential component for the normal development of both hair cells and photoreceptor cells.

**Figure 1:**
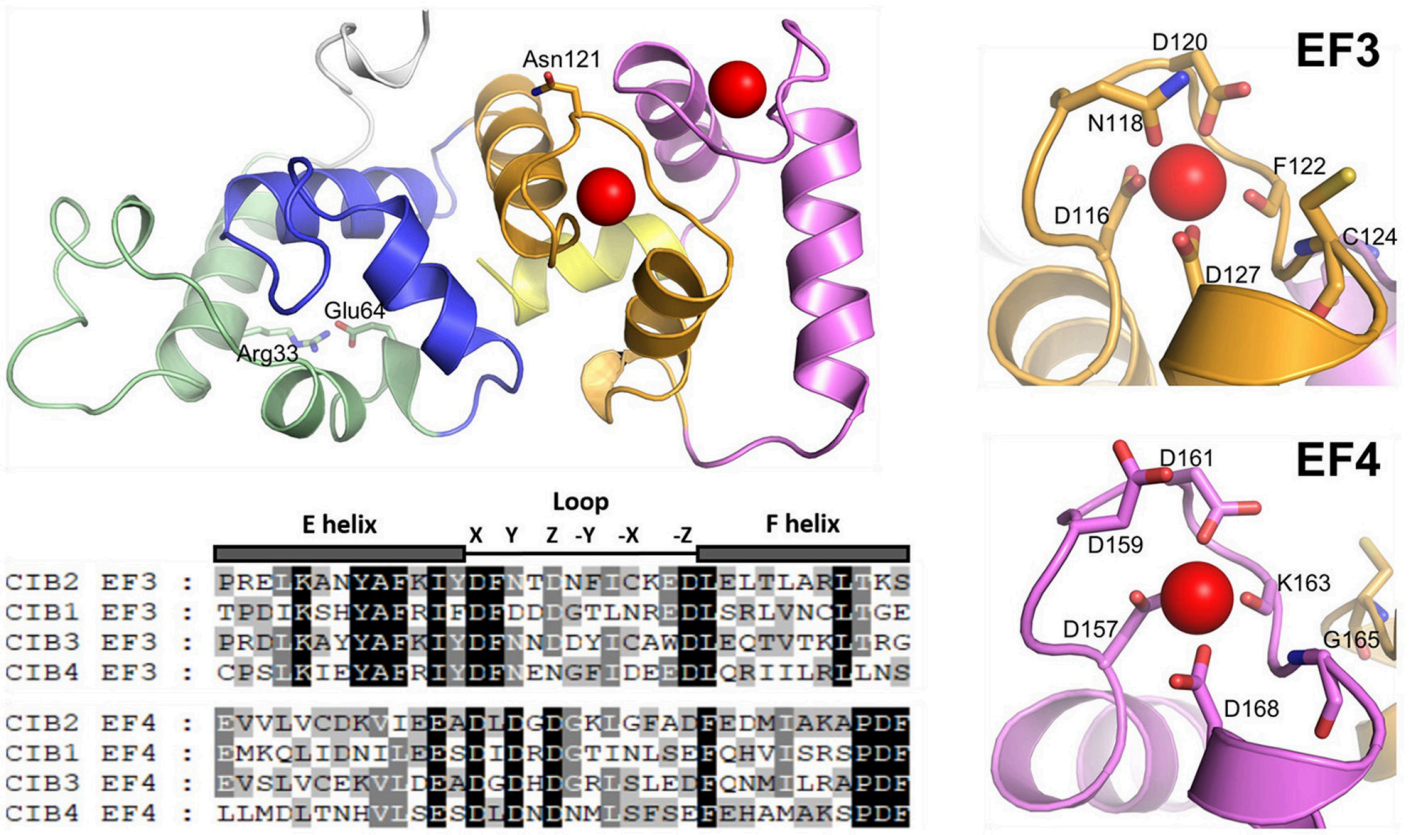
Cartoon representation of monomeric WT CIB2 in its Ca^2+^-bound form. The homology model is based on the X-RAY structure of Ca^2+^-bound CIB1 (see section Materials and Methods). The N-terminal region is colored gray, while the C-terminal region (helix 10) is colored yellow. EF1, EF2, EF3, and EF4 are colored green, blue, orange, and magenta, respectively. Asn 121 and Glu 64, found to be involved in an allosteric connection, are represented by sticks, as well as Arg 33, electrostatically interacting with Glu 64. The sequence alignment of CIB2 with CIB1, CIB3, and CIB4 is also shown, restricted to the metal-coordinating EF-hands, namely EF3 and EF4. Amino acids contributing to the coordination of Ca^2+^ according to the canonical pentagonal bipyramid geometry are marked by their respective position and labeled in the zoomed-in protein cartoon on the right.

Among the members of the CIB family, CIB1 is the protein that has been characterized in deeper biochemical and structural detail (Yamniuk et al., 2004, 2006, 2007; Gentry et al., 2005; Yamniuk and Vogel, 2005; Huang et al., 2011). CIB1 and CIB2 are homologous proteins, but both the sequence identity (37.4%) and the overall similarity (60%) are not extremely high. Differences are found throughout the primary structure and importantly, key residues are substituted in the metal-ion binding sites EF3 and EF4 (Figure 1). These differences may be reflected in an overall distinct structure/function behavior of CIB2 as compared to CIB1. To date, only few studies have focused on the biochemical and biophysical characterization of CIB2 (Häger et al., 2008; Blazejczyk et al., 2009; Huang et al., 2012) and a comprehensive picture that allows a molecular-level understanding of its biological properties under physiological conditions as well as their alteration in USH1J is currently missing.

In this work, we present an exhaustive characterization of human wild type (WT) and E64D CIB2 by using an integrated biochemical and biophysical approach to highlight the molecular defects of the variant associated with USH1J. Interestingly, we found that CIB2 does not possibly work as a calcium sensor under physiological conditions, because its affinity for Ca^2+^ is too low for normal intracellular levels. Instead, CIB2 has a fairly high affinity for Mg^2+^ and it is probably Mg^2+^-bound under physiological conditions. At odds with CIB1, which is monomeric both when isolated and when interacting with its target (Gentry et al., 2005), we found that CIB2 is a non-covalent dimer under conditions that mimic the physiological ones, and as such it interacts with its physiological target α7B integrin. NMR spectroscopy revealed a long range allosteric communication between the residue E64, located at the N-terminal domain, and the metal cation binding site EF3, located at the C-terminal domain (Figure 1). The E64D mutation associated with USH1J, although conservative, apparently breaks up such inter-domain communication resulting in the impaired ability of CIB2 to switch to its Mg^2+^ (and Ca^2+^)-bound form, thus suggesting that the molecular defect associated with CIB2 and causing USH1J resides in its inability to sense Mg^2+^ and adopt the required conformation.

## Materials and methods

### Materials

QuikChange II Site-Directed Mutagenesis kit was purchased from Agilent. The Bradford reagent was purchased from Bio-Rad. Chromatographic columns were purchased from GE Healthcare, and synthetic oligonucleotides were from Eurofins. All other chemicals were purchased from Sigma Aldrich. All purchased chemicals were of the highest commercially available purity grade.

### Expression and purification of human wild type and E64D CIB2

The cDNA of human CIB2 isoform 1 (Uniprot entry 075838-1) was cloned into a pET24a(+) vector (Genscript) containing a 6xHis-tag at the N-terminal, followed by Tobacco Etch Virus (TEV) cleavage site. The plasmid was used to transform BL21 DE3 cells. Cells were grown in LB medium or in M9 minimal medium supplemented with ^15^NH_4_Cl (1 g L^−1^) as a sole nitrogen source for NMR studies, at 37°C until the OD_600_ reached a value around 0.4. Flasks were then cooled down and, after induction by 0.5 mM ITPG at OD_600_ = 0.6, bacteria were grown at 15°C for 20 h. After centrifugation at 5,500 g (20 min at 4°C) the obtained pellets were suspended in lysis buffer (20 mM TRIS pH = 7.5, 0.5 M NaCl, 20 mM imidazole, 1 mM DTT, 5 U/mL DNAse, 0.1 mg/mL lysozyme, 1 mM PMSF, 2.5 mM MgCl_2_) and incubated at 25°C for 30 min. In addition, 10–12 sonication cycles on ice, 10 s each, were performed. Soluble and insoluble fractions were separated by centrifugation at 16,000 g, 4°C for 30 min. WT CIB2 was found in high amount in the soluble phase and it was directly leaded in a 5 mL His-trap FF Crude column (GE Healthcare) previously equilibrated with loading buffer (20 mM TRIS, pH = 7.5, 0.5 M NaCl, 1 mM DTT, 20 mM imidazole). A one-step elution by 500 mM imidazole was chosen after initial tests with a gradient. In order to remove the imidazole excess to allow for TEV-protease activity, His-CIB2 was dialyzed against 50 mM TRIS pH 8, 150 mM NaCl, 1 mM DTT and then incubated with a previously prepared His-tagged TEV-protease (Dal Cortivo et al., 2018) (ratio 1:30) overnight at 8°C. Tag-free CIB2 was purified from His-TEV and cleaved His-tails by reloading into a His-trap column and collecting the flow-through. Protein concentration was measured by a Bradford assay optimized for homolog calcium sensor proteins or by using the predicted molar extinction coefficient (ε_280_ = 6,400 M^−1^cm^−1^, http://protcalc.sourceforge.net/) and the purity was verified by SDS-PAGE to be at least 90%. Purified WT CIB2 was washed in 20 mM TRIS pH 7.5, 150 mM KCl, 1 mM DTT, using an Amicon concentrator. Protein aliquots were then flash-frozen and stored at −80°C until use.

The E64D point mutation was obtained by site-directed mutagenesis on the complete cDNA of CIB2 using a forward primer (5′-ATCATTCAAATGCCGGACCTGCGTGAGAACCCGTT-3′) and a reverse primer (5′-AACGGGTTCTCACGCAGGTCCGGCATTTGAATGAT-3′). Protein expression was performed as for the WT but the mutant protein was found to concentrate in the insoluble fraction, thus requiring purification from the inclusion bodies. After cell lysis, the insoluble pellets were suspended in the unfolding buffer (20 mM TRIS pH 7.5, 0.5 M NaCl, 6M guanidine hydrochloride, 20 mM imidazole, 1 mM DTT) and incubated overnight at 4°C. Unfolded CIB2 was loaded into a His-trap column and then renatured by a gradient from 0 to 100% of refolding buffer (unfolding buffer guanidine hydrochloride-free) setting the flow rate to 1 mL/min (100 mL total volume). After elution with 500 mM imidazole E64D CIB2 was treated as the WT protein.

### Peptides

The peptide corresponding to the membrane proximal segment of the cytoplasmic domain of α7B integrin (Uniprot entry: Q13683) comprised between residues 1101–1116 (α7B_M, Ac-LLLWKMGFFKRAKHPE-NH_2_) and a scrambled peptide obtained by shuffling the α7B_M sequence (Scrb, Ac-KEFWGLHAKPRLKLMF-NH_2_) were synthesized by GenScript USA Inc. (New Jersey, 144 USA). The purity of peptides, estimated by HPLC, was ≥95% and concentration was determined using the predicted molar extinction coefficient (ε_280_ = 5,690 M^−1^cm^−1^, http://protcalc.sourceforge.net/).

### Circular dichroism spectroscopy and thermal denaturation profiles

Secondary and tertiary structures of WT and E64D CIB2 and thermal denaturation profiles were investigated by using a Jasco J-710 spectropolarimeter equipped with a Peltier type cell holder, using protocols previously described (Astegno et al., 2014; Marino et al., 2014, 2015a,b; Vocke et al., 2017). Briefly, near UV (320–250 nm) and far UV (250–200 nm) spectra of ~30 μM and 12 μM CIB2 respectively were collected at 37°C after consecutive additions of 0.5 mM EDTA, 1 mM Mg^2+^ and 1 mM Ca^2+^. Quartz cuvettes were used both for near UV (1 cm) and far UV (0.1 cm). Solvent spectra were recorded and considered as a blank.

Thermal denaturation profiles were collected in the same conditions as for far UV spectra by monitoring ellipticity signal at 222 nm in a temperature range between 4 and 70°C (scan rate 90°C/h).

Titration experiments were designed starting from the apo WT and E64D CIB2 in order to estimate an apparent *K*_*d*_ value (K_d_^app^) for calcium and magnesium binding, similar to what was done previously for calmodulin (Maune et al., 1992). The dichroic signal (in terms of molar ellipticity per residue (MRE) at Θ = 222 nm, indicative of typical acquirement of secondary structure, was followed as a function of the concentration of free Mg^2+^ or Ca^2+^. In order to obtain a controlled free ion concentration under well-defined pH and salt conditions, the MaxChelator software (http://maxchelator.stanford.edu/) was used. Each titration point represents an independent sample where 0.5 μL of a Ca^2+^ or Mg^2+^ stock solution at the appropriate concentration was added to the fixed volume (200 μL) of 12 μM CIB2 aliquots in the presence of 1 mM EGTA. After 3 min incubation at 25°C, three replicas of each spectrum were collected.

### Fluorescence spectroscopy

Fluorescence spectra were obtained with a Jasco FP750 spectrofluorimeter. The interaction of WT CIB2 with α7B_M and Scrb peptides and that of E64D with α7B_M was studied by monitoring the peptide intrinsic fluorescence. The only Trp residue of both peptides was selectively excited at 295 nm and fluorescence emission was recorded from 300 to 400 nm, setting 5 nm excitation and emission bandwidths. Two scan averaged spectra were recorded. α7B_M (4 μM) was titrated with increasing concentrations of WT or E64D CIB2 in 20 mM Hepes, 150 mM KCl, 1 mM DTT pH 7.5 at 37°C in the presence of 1 mM Mg^2+^ and 1 mM Ca^2+^. Titration experiments were performed by monitoring the change (blue-shift) in wavelength (λ) of the peptide emission peak on the fluorescence spectrum. The apparent equilibrium dissociation constant (*K*_*d*_) was calculated by using the following equation:

(1) $$y\;=\;y_{0}+ax/(K_{d}+x)$$

where *y*_0_ is the wavelength of the peptide emission peak in the absence of WT/E64D CIB2, *a* is the difference between the maximum and minimum (1/λ) × 10^5^ values of the peptide emission peak as a function of *x*, the concentration of CIB2.The intrinsic fluorescence emission of the single Trp of the Scrb peptide (4 μM) was measured in the same buffer at 37°C, in the presence of 2, 4, and 8 μM WT CIB2.

8-Anilinonaphthalene-1-sulfonic acid (ANS) fluorescence was used to probe the changes in hydrophobicity of WT and E64D CIB2 upon Mg^2+^ and Ca^2+^ binding. Two micromolar of WT or E64D CIB2 in 20 mM Hepes pH 7.5, 150 mM KCl, 1 mM DTT, was incubated with 30 μM ANS and fluorescence was measured after the addition of 0.5 mM EDTA, 1 mM Mg^2+^ and 1 mM Ca^2+^. ANS fluorescence spectra were recorded at 37°C in the 400–650 nm range after excitation at 380 nm, with 5 nm bandwidths. Three scan averaged spectra were recorded.

### Size exclusion chromatography

The molecular weight (MW) of the Ca^2+^-free, Mg^2+^-bound and Mg^2+^/Ca^2+^ -bound states of WT and E64D CIB2 was determined by size exclusion chromatography (SEC) in an ÄKTA FPLC system using a Superose 12 column (10/300GL, GE Healthcare). Standard proteins for calibration were: carbonic anhydrase (29 kDa), alcohol dehydrogenase (150 KDa), β-amylase (200 kDa), and cytocrome c (12.4 kDa). The column was equilibrated with a buffer containing 20 mM Tris pH 7.5, 150 mM KCl, 1 mM DTT with either 3 mM EGTA or 2 mM EGTA + 3 mM Mg^2+^ or 3 mM Mg^2+^ + 2 mM Ca^2+^ added. WT (100 μM) or E64D (70 μM) CIB2 were incubated with 3 mM EGTA or 2 mM EGTA + 3 mM Mg^2+^ or 3 mM Mg^2+^ + 2 mM Ca^2+^ at 25°C for 5 min before being applied to the column. The protein elution profile was recorded at 280 nm; elution volumes *V*_*e*_ were determined and the distribution coefficient *K*_*d*_ was calculated according to the equation:

(2) $$K_{d}=(V_{e}-V_{0})/(V_{t}-V_{0})$$

in which *V*_*t*_ is the total column volume and *V*_0_ is the void volume. Molecular weights were determined from a calibration plot of log(MW) vs. *K*_*d*_.

### Native page

In order to investigate CIB2 oligomeric state with another approach, the Ferguson plot technique (Ferguson, 1964) was used. Three continuous gels (lacking a stacking phase) under non-denaturing conditions were polymerized at increasing acrylamide concentration (10%, 12%, 15%) using two different BSA concentrations as standards (0.25, 0.41 mgmL^−1^). Twenty micromolar of CIB2 was incubated at room temperature with EGTA (4.5 mM), Mg^2+^ (3 mM EGTA + 4.5 mM Mg^2+^) or both Ca^2+^ and Mg^2+^ (3 mM Ca^2+^, 4.5 mM Mg^2+^) in the presence of 1 mM DTT for 20 min. Samples were loaded in each gel and let run as in a normal electrophoresis experiment for 40 min, 200 V at room temperature. Bands were visualized by Comassie Blue staining. Data analysis was performed as explained in (Ferguson, 1964).

### Dynamic light scattering

Dynamic light scattering (DLS) measurements were performed with a Zetasizer Nano-S (Malvern Instruments) and polystyrene low volume disposable sizing cuvettes (ZEN0112) using a general setup optimized previously (Sulmann et al., 2014; Marino et al., 2015a, 2017; Vocke et al., 2017). Viscosity and refractive index were set to 0.6864 cP and 1.33 (default values for water), respectively; the temperature was set to 37°C, with 2 min equilibration time. The measurement angle was 173° backscatter, and the analysis model was set to multiple narrow modes. For each measurement, 12 determinations were performed, each consisting of 14–16 repetitions. DLS measurements were performed on the samples of dimeric WT or E64D CIB2 in 20 mM Tris–HCl pH 7.5, 150 mM KCl, 1 mM DTT with 3 mM EGTA or 2 mM EGTA + 3 mM Mg^2+^ or 3 mM Mg^2+^ + 2 mM Ca^2+^, immediately after their purification by SEC. Each measurement was run for 5 h. The samples were filtered through an Anotop 10 filter (Whatman, 0.02 μm) before each measurement.

### Nuclear magnetic resonance experiments and data analysis

NMR spectra were acquired on a Bruker Avance III spectrometer (Bruker, Karlsruhe, Germany) operating at 600.13 MHz proton Larmor frequency, and equipped with a cryogenic probe. The spectra were recorded at 25°C, the samples were at protein concentration of 320 μM (unless otherwise specified) in 20 mM Hepes, 100 mM KCl, 1 mM DTT, pH 7.5 and 7% D_2_O.

A standard ^1^H-^15^N heteronuclear single-quantum coherence (HSQC) pulse sequence was used, with pulsed field gradients for suppression of the solvent signal and cancellation of artifacts. ^1^H-^15^N HSQC spectra were acquired with a data matrix consisting of 2K (F2, ^1^H) × 256 (F1, ^15^N) complex points, spectral windows of 8417.509 Hz (^1^H) × 2189.44 Hz (^15^N), 8 transients, and 1.5 s relaxation delay.

NMR titration experiments were run on 320 μM ^15^N-WT CIB2 with Ca^2+^ ion added stepwise from a concentrated stock solution. The following protein/ligand ratios were analyzed by ^1^H-^15^N HSQC spectra: 1:1, 1:3, 1:5, 1:7, 1:10, 1:15, 1:20. Intensity perturbations were computed as: I/I_max_, where I is the signal intensity at titration step analyzed, and I_max_ is the maxium signal intensity at the last titration point.

The K_d_ values were obtained by fitting the NMR isotherms to a singlestep one-site binding model (Equation 3: Figures **5D,F**) or to a single step with Hill slope binding model (Equation 4: **Figure 5E**), using GraphPad software according to the following equations:

(3) $$\begin{array}{l} I/I_{max}=((K_{d}+{\left[L\right]}_{t}+{\left[P\right]}_{t})-((K_{d}+{\left[P\right]}_{t}\\ \;+{\left[P\right]}_{t}{)}^{2}-4{\left[L\right]}_{t}{\left[P\right]}_{t}{)}^{0.5})/2{\left[P\right]}_{t}; \end{array}$$

(4) $$I/I_{max}=B_{max}{\left[L\right]}^{h}/(K_{d}^{apph}+{\left[L\right]}^{h})$$

where *I/I*_*max*_ is the relative intensity observed at each titration point, *[P]*_*t*_ and *[L]*_*t*_ represent the total protein and ligand concentration, respectively, *[L]* is approximated by *[L]*_*t*_ and *K*_*d*_ is the dissociation constant of the complex.

For Equation (4) K_d_^app^ is the apparent equilibrium dissociation constant, h is the Hill slope, and *B*_*max*_ is the maximum intensity observable. All data were processed and analyzed using TOPSPIN 3.2 (Bruker, Karlsruhe, Germany) and CARA software.

### Building of WT CIB2 homology model

The homology model of WT CIB2 monomer was built using the MODWEB-MODBASE server version r189 (Pieper et al., 2014). Briefly, 4 out of 189 structural models were selected based on the MPQS, TSVMOD, LONGEST_DOPE and DOPE criteria. The most reliable model, covering the 13–187 region of the full protein sequence, was built based on the X-ray structure of human CIB1 [PDB entry: 1XO5 (Gentry et al., 2005) chain A], which shares 39% sequence identity with CIB2. Ca^2+^ ions were manually positioned in EF3 and EF4 binding sites based on the experimental coordinates of the Ca^2+^-loaded CIB1 structure (1XO5.pdb) (Gentry et al., 2005). The structure was energy-minimized in two steps, first with the steepest descent and then with the conjugate gradients algorithm, keeping in both cases the position of the backbone atoms restricted, according to a previous protocol used for other Ca^2+^ sensor proteins (Marino et al., 2015b; Marino and Dell'Orco, 2016).

## Results

### Wild-type and E64D CIB2 form non-covalent dimers with different colloidal properties

We investigated the oligomeric state of both WT and E64D CIB2 by three different approaches, namely PolyAcrylamide Gel Electrophoresis under non-denaturing conditions (native-PAGE), analytical SEC and DLS. Figure S1a shows that, under denaturing conditions (SDS-PAGE), the electrophoretic mobility of CIB2 is compatible with that of a 21.6 kDa protein, although the band is shifted to a slightly higher molecular weight, as previously observed in other studies (Blazejczyk et al., 2009; Huang et al., 2012). Gels obtained under non-denaturing conditions show that in the presence of a reducing agent (1 mM DTT) both apo (lane 2) and Ca^2+^-bound (lane 4) CIB2 run as single bands (Figure S1b). However, multiple bands were observed in the absence of DTT independent of the presence of Ca^2+^ (lanes 1 and 3). This is compatible with the formation of covalent oligomers due to disulfide bridges resulting from the oxidation of thiol groups in either of the four Cys residues.

In order to investigate the nature of the single bands observed in the native-PAGE experiments, we performed analytical SEC of both WT and E64D CIB2 in the apo form as well as in the presence of Mg^2+^ and Ca^2+^/Mg^2+^ and determined the molecular weight (MW) by using a calibration curve shown in Figure S2. As previously observed for other Ca^2+^/Mg^2+^ sensor proteins (Sulmann et al., 2014; Marino et al., 2015a; Astegno et al., 2016, 2017; Vallone et al., 2016) the elution profile was sensitive to the metal ion loading state (Figure S2) and resulted in an apparent lower MW for the Ca^2+^/Mg^2+^ bound form of CIB2 compared to the apo-form (Table S1). Under reducing conditions, the elution profiles for both WT and E64D CIB2 variants in the Ca^2+^/Mg^2+^ form (MW = 37–39 kDa, Table S1) were compatible with a dimer and incompatible with a monomer (MW~22 kDa). The dimeric nature of CIB2 under all the tested conditions was further confirmed by Ferguson plots, which estimated a MW in the 51–53 kDa range for both WT and E64D variants independent on the cation loading state (Table S1).

**Figure 2:**
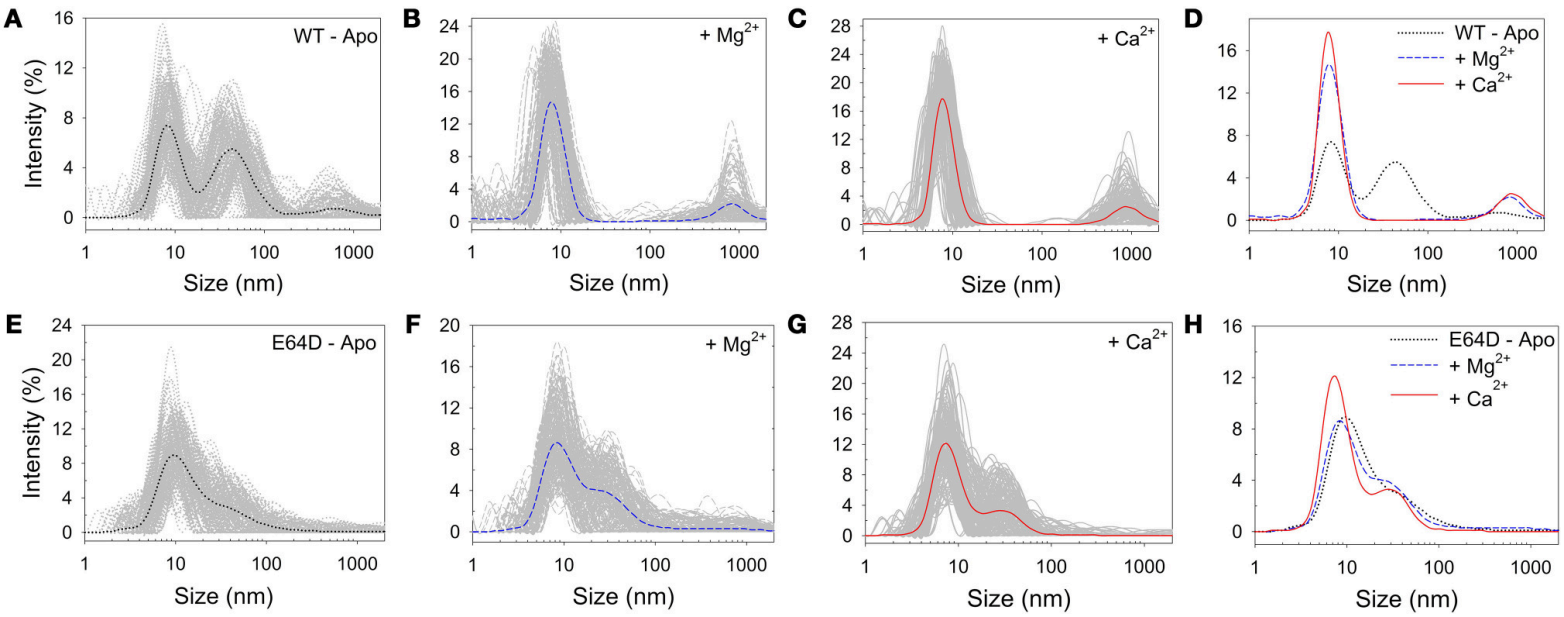
Dynamic light scattering spectroscopy. Comparison between hydrodynamic diameters measured (T = 37°C) for WT **(A–D)** and E64D **(E–H)** CIB2 in the presence of: 3 mM EGTA **(A,E)**, 3 mM Mg^2+^ + 2 mM EGTA **(B,F)**, 3 mM Mg^2+^ + 2 mM Ca^2+^ **(C,G)**. **(D,H)** Superimposition of the average curves obtained by **(A–C)** for the WT **(D)** and **(E–G)** for the E64D CIB2 **(H)**.

Samples from SEC experiments were further analyzed by DLS immediately after elution. Results are reported in Figure 2. The DLS intensity profile of WT CIB2 in the absence of Ca^2+^ and Mg^2+^ showed multiple peaks and a generally high polydispersity (Figure 2A), with two not-well separated prevailing peaks. However, the addition of Mg^2+^ (Figure 2B) or Ca^2+^ (Figure 2C) led to a general improvement of the colloidal properties and a single prominent peak was distinguished in both cases, which allowed the determination of the hydrodynamic diameter (*d*^*Mg*^ = 8.43 ± 0.12 nm and *d*^*Ca*^ = 8.18 ± 0.01 nm, respectively; see Figure 2D). The E64D CIB2 variant instead showed less satisfactory colloidal properties, as under no tested condition, apo (Figure 2E), Mg^2+^ (Figure 2F), or Ca^2+^ (Figure 2G) could a single, prevailing peak be observed in the intensity profile. The constant presence of higher-size aggregates and the overlapping of peaks (Figure 2H) prevented an estimate of the hydrodynamic diameter to be made for this CIB2 variant.

The differences observed in the DLS profiles of WT and E64D CIB2 under the tested conditions prompted us to analyze the time-dependent properties of the dispersions. The mean count rate (MCR) of the samples, which can be indicative of time-dependent protein aggregation, was thus followed over time for 5 h (Figure S3). Interestingly, for WT CIB2 (Figure S3a) no trend was observed under the investigated conditions, but significant fluctuations in the MCR were observed especially in the apo conditions (150–600 kcps), in line with a partly reversible protein aggregation process. Less prominent but still significant MCR fluctuations were observed in the presence of Ca^2+^ or Mg^2+^ (150–300 kcps). A somewhat different pattern was detected for E64D CIB2 (Figure S3b). Both in apo conditions and in the presence of Ca^2+^ and Mg^2+^ a slow, constantly increasing trend in MCR was observed. In the sole presence of Mg^2+^, significantly broad fluctuations of MCR (150–400 kcps) were detected, which also showed a slowly increasing trend.

### Apo, Mg^2+^- and Ca^2+^-loaded WT and E64D CIB2 show different levels of folding

**Figure 3:**
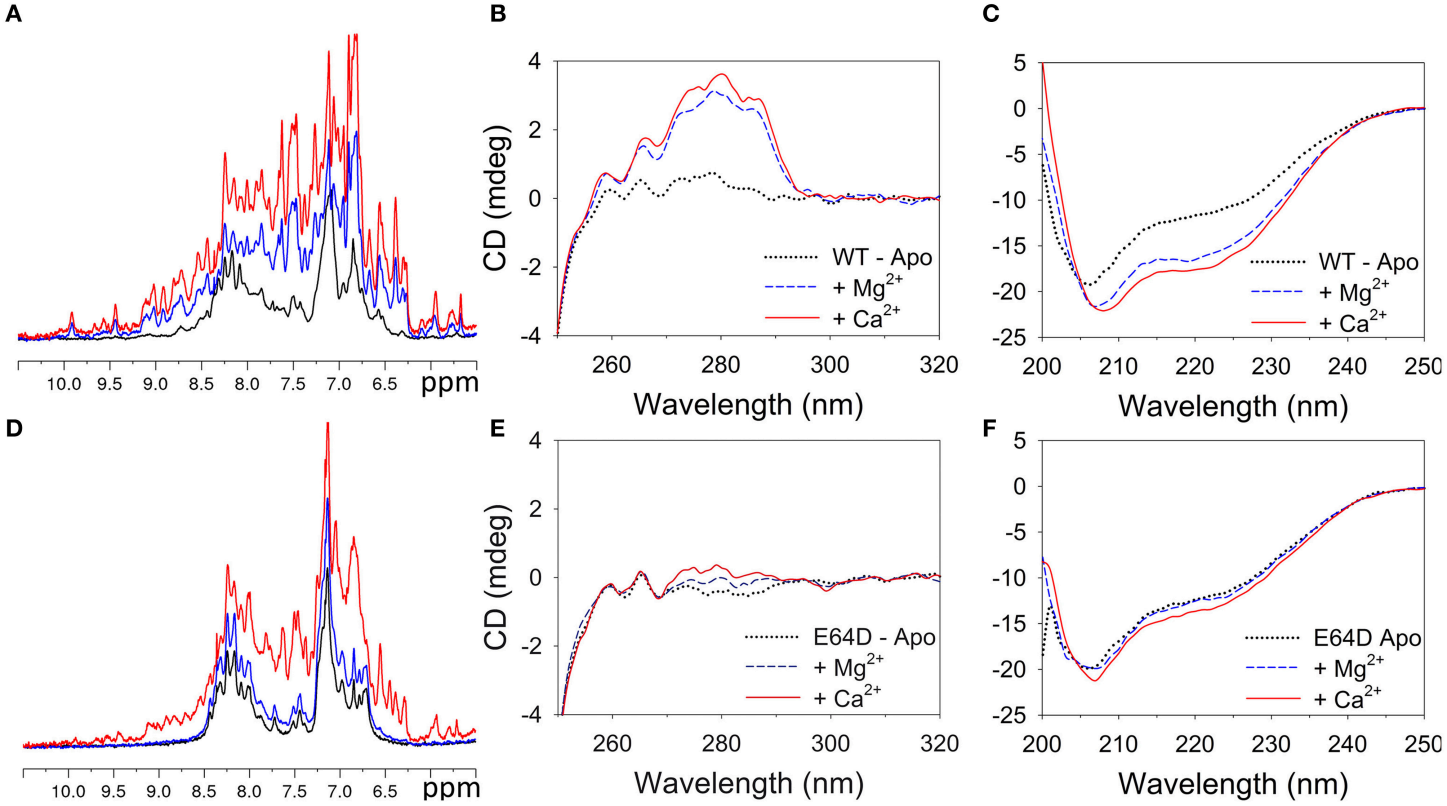
Conformational changes in WT and E64D CIB2 upon interaction with Mg^2+^ and Ca^2+^. **(A–D)** Superimposition of the ^1^H NMR spectra of WT **(A)** and E64D **(D)** CIB2 in their apo form (black), and in the presence of 1 mM Mg^2+^ (blue) and 1 mM Mg^2+^ + 1 mM Ca^2+^ (red). Both unlabeled proteins were at a 50 μM concentration. The spectra were recorded at 600 MHz and 25°C. The intensity of the spectra has been rescaled for better spectra visualization. **(B,C,E,F)** Near UV spectra of _~_28 μM WT CIB2 **(B)** and 30 μM E64D CIB2 **(E)** and far UV spectra of 12 μM WT CIB2 **(C)** and E64D CIB2 **(F)** after sequential additions of 0.5 mM EDTA (black dotted), 1 mM Mg^2+^ (solid blue lines) and 1 mM Ca^2+^ (red solid lines). Temperature was fixed at 37°C, each spectrum represents the mean of 5 accumulations.

One-dimensional (1D) ^1^H NMR spectroscopy and far/near UV CD spectroscopy were used to monitor the folding state of WT and E64D CIB2 under different conditions (Figure 3).

1D ^1^H NMR spectroscopy is a fast and powerful technique that can provide information on the global fold of a protein. In 1D ^1^H NMR spectra, the signal dispersion in the regions of the amide (6–10 ppm), and methyl (−0.5 to 1.5 ppm) protons provides indications on the folded globular state of the proteins. Moreover calcium binding proteins show typical downfield-shifted NMR peaks at ~10.5 ppm belonging to residues of the EF-hands upon binding of divalent metals (Huang et al., 2012). The 1D ^1^H NMR spectrum of WT CIB2 in the absence of metal ions displayed evidence of chemical shift dispersion but also line broadening in the amide region (Figure 3A, black line). Addition of 1 mM Mg^2+^ promoted a large change in the WT CIB2 spectrum (Figure 3A, blue line); the signals appeared more disperse and sharp indicating the ability of the protein to assume a globular folded structure upon binding of the metal cation. Further addition of 1 mM Ca^2+^ (Figure 3A, red line) caused slight changes in the 1D ^1^H NMR spectrum, indicating a low degree of structural rearrangement upon binding of the second ion.

The NMR data recorded on samples of E64D CIB2 drive to different conclusions. The 1D ^1^H NMR spectrum of the E64D CIB2 in the absence of metal ions displayed narrow signal dispersion throughout the spectrum and especially in the region of amide protons (Figure 3D, black line). The addition of 1 mM Mg^2+^ (Figure 3D, blue line) did not promote changes in the NMR spectrum clearly indicating the inability of the E64D CIB2 to bind Mg^2+^. Upon subsequent addition of Ca^2+^ the NMR signals appeared more disperse as a consequence of the binding of the metal ion, however the 1D spectrum suggests that the protein still retains a certain degree of flexibility and it is not characterized by a rigid tertiary structure (Figure 3D, red line).

Near UV (250–320 nm) CD spectroscopy provides information as to the microenvironment of the aromatic amino acids Phe, Tyr, and Trp, which contribute to the stabilization of protein tertiary structure. CIB2 lacks Trp, therefore near UV CD spectra represent a fingerprint of the possible variations in tertiary structure in the Tyr (5 residues) and Phe (16 residues) microenvironments upon metal cation binding. Monitoring the CD signal in the far UV region (200–250 nm) provides instead information as to variations of the protein secondary structure. The near UV CD spectrum of WT CIB2 in the absence of metal cations was almost flat (Figure 3B, black dotted line), nevertheless some helical content was clearly observed in the far UV region (Figure 3C), thus suggesting that apo CIB2 forms a molten globule state, in line with NMR findings. Addition of 1 mM Mg^2+^ led to a significant response both in the Phe and Tyr bands (Figure 3B, blue dashed line) and to a remarkable increase in the helical content as observed in the far UV region (Figure 3C). Notably, upon addition of physiological concentrations of Mg^2+^ the far UV spectrum acquired the typical α-helix minima at 208 and 222 nm, while the first minimum was shifted to 206 nm in the apo form (black dotted line). Further addition of 1 mM Ca^2+^ refined the near UV CD spectrum especially in the Tyr region (Figure 3B, solid red line) and further increased the intensity of the far UV spectrum (Figure 3C).

The behavior of E64D CIB2 was substantially different. When exposed to the same Mg^2+^ and Ca^2+^ conditions, E64D CIB2 showed only minor variations in the near UV region, in line with a substantial conservation of the molten globule conformation independent of the metal cation (Figure 3E). Only a slight response to Ca^2+^ was observed in the far UV region (Figure 3F), however the first minimum at 206 nm did not shift to 208 nm upon addition of Mg^2+^ or Ca^2+^, at odds with the WT variant.

### Hydrophobicity and thermal denaturation profiles of WT and E64D CIB2

In their apo form, both WT and E64D CIB2 present a partially folded, molten globule conformation, thus suggesting a significant solvent-exposition of hydrophobic patches. We investigated the surface hydrophobicity of CIB2 by using the fluorescent probe ANS, whose fluorescent properties will change as it binds to hydrophobic regions on the protein surface. Results are shown in Figure S4. Both ANS fluorescence spectra of WT and E64D CIB2 highlight a significant hydrophobicity of the protein surface under all the tested conditions, as clearly displayed by the remarkable blue-shift of the fluorescence emission maximum (27 nm for WT CIB2, 31 nm for E64D CIB2, in the apo state, Table S2) and the relative increase in fluorescence intensity as compared to the emission of ANS alone (2.6- to 2.8-fold, in the apo state, Table S2). Addition of 1 mM Mg^2+^ or 1 mM Ca^2+^ slightly reduced the blue-shift (1–2 nm, depending on the CIB2 variant, Table S2), however the change in relative fluorescence (F^max^/F^ref^) was higher for WT (2.1–2.2 vs. 2.6) than for E64D CIB2 (2.5–2.6 vs. 2.8). Overall, fluorescence data confirm that both WT and E64D CIB2 are highly hydrophobic and the pathogenic variant maintains higher hydrophobicity in the presence of Mg^2+^ or Ca^2+^ compared to the WT.

**Figure 4:**
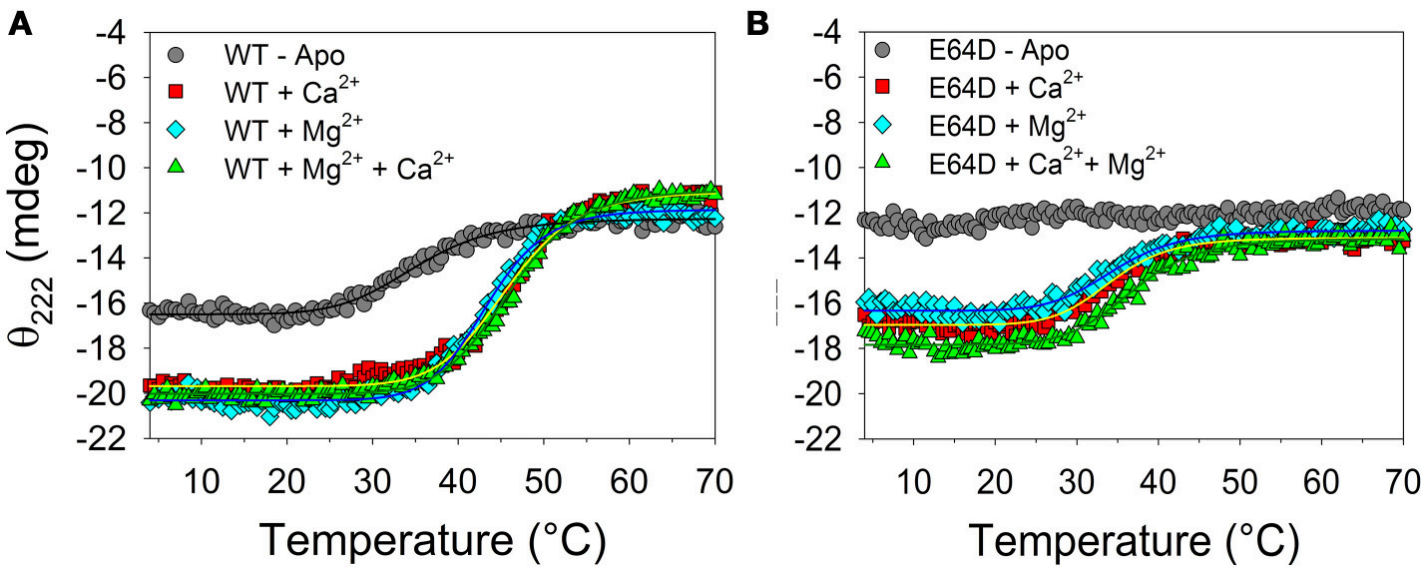
Thermal denaturation of WT and E64D CIB2. Thermal denaturation profiles were recorded between 4 and 70°C with 12 μM WT CIB2 **(A)** and E64D CIB2 **(B)** in the presence of 0.5 mM EDTA (gray circles), 1 mM Ca^2+^ (red squares), 1 mM Mg^2+^ (light blue diamonds) and 1 mM Ca^2+^ + 1 mM Mg^2+^ (green triangles). Data fitting was performed using a Hill 4 parameter function, results are shown by solid lines and parameters are reported in Table S2.

The thermal stability of WT and E64D CIB2 in the 4–70°C range was investigated by monitoring the dichroic signal at 222 nm, where a minimum was observed in the far UV CD spectrum (Figure 3). Thermal denaturation profiles are reported in Figure 4. Apo WT CIB2 was found to be rather unstable, having a melting temperature of 35.1°C (Table S2). Addition of Mg^2+^ increased the thermal stability of ~11°C, and a similar effect was observed for addition of 1 mM Ca^2+^, although the stabilization was lower (~8°C). In the presence of both cations the thermal stability resembled that of Mg^2+^ (T_m_ = 45.9°C, Table S2). The transition was faster in the metal-bound states compared to the apo-state (H_c_ = 11–12.5 vs. 7.5, Table S2). The persistent CD signal at 222 nm (Figure 4A) suggests that the transition ended in a still partially folded structure, independent of the presence of Mg^2+^ or Ca^2+^. Interestingly, the thermal profile of apo E64D CIB2 was unperturbed in the scanned range of temperature, as no transition was observed (Figure 4B). Addition of Mg^2+^ resulted in a T_m_-value ~ 11.5°C lower than that of WT CIB2, and a very similar effect was observed after the addition of 1 mM Ca^2+^ (Table S2). Only the co-presence of both cations slightly increased the thermal stability of E64D CIB2, which however was ~9°C lower than the respective WT case (Table S2). All the transitions observed for E64D CIB2 were significantly slower compared to the respective cases in WT CIB2 (compare the H_c_ values, Table S2).

### Mg^2+^ and Ca^2+^ binding to WT CIB2 explored by NMR spectroscopy reveals an inter-domain allosteric switch

Two-dimensional ^1^H-^15^N HSQC NMR spectra are often employed to investigate protein structural changes. The NMR chemical shift of the signals belonging to all HN groups of the protein is a sensitive reporter of the local and global structure rearrangements occurring upon ligand binding events. The NMR signals of the ^1^H-^15^N HSQC spectrum of the apo ^15^N WT CIB2 were broad and poorly dispersed in line with the previous observation that apo CIB2 forms a molten globule state.

**Figure 5:**
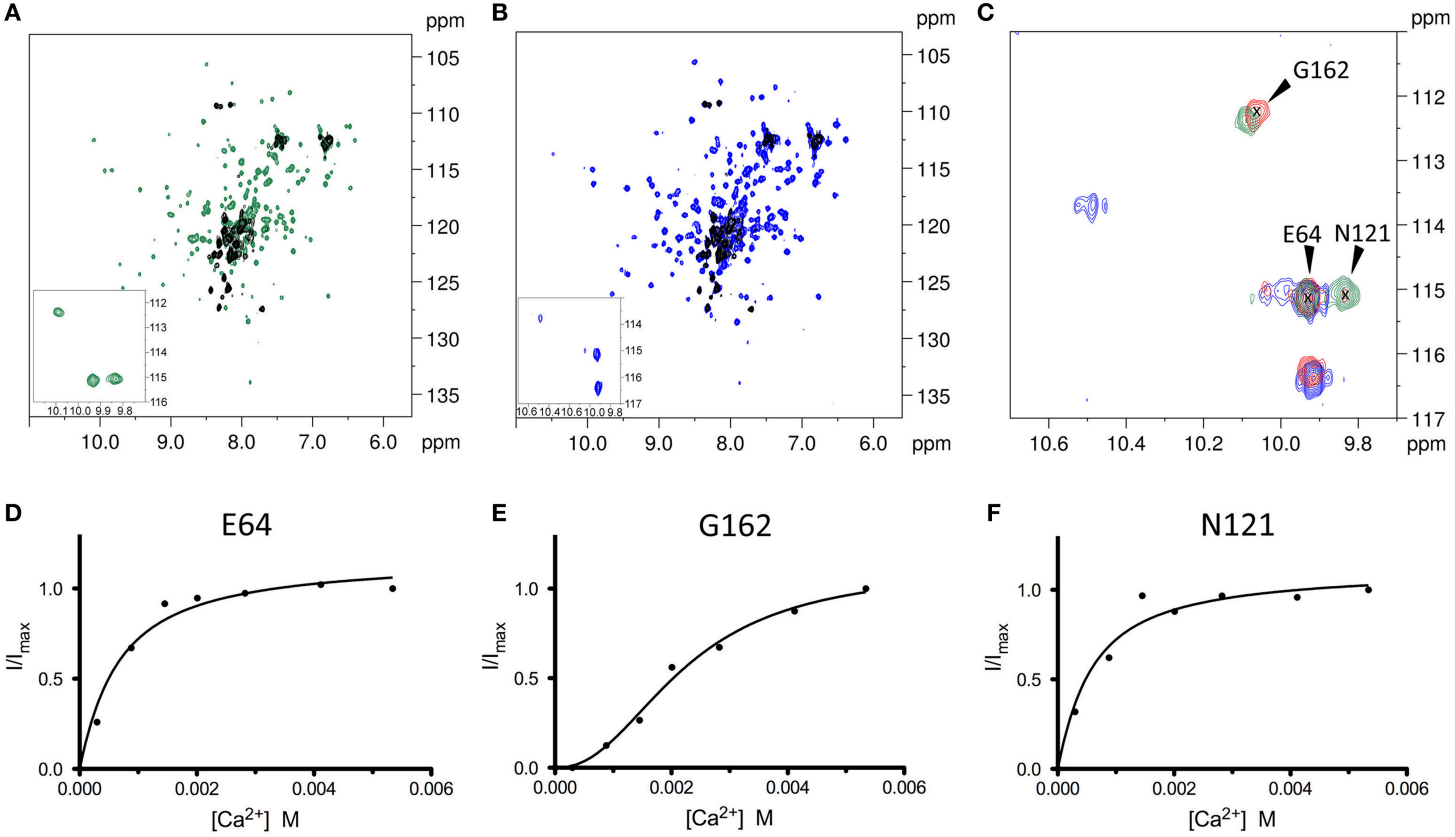
^1^H-^15^N HSQC NMR spectra of WT CIB2 in its apo form and in the presence of Mg^2+^ and Ca^2+^ highlight an allosteric communication between E64 and N121. **(A)** Superimposition of the two-dimensional ^1^H-^15^N HSQC NMR spectra of the apo- (black), and Ca^2+^-bound (green) ^15^N-WT CIB2. **(B)** Superimposition of the HSQC spectra of apo (black), and Mg^2+^-bound (blue) ^15^N-WT CIB2. In the insets, zoom of the HSQC spectra of the downfield peaks of the metal-bound forms of ^15^N-WT CIB2. Metal ions were present at a protein:metal ratio of 1:15. **(C)** Superimposition of downfield region of the ^1^H-^15^N HSQC NMR spectra recorded on ^15^N-WT CIB2 containing 15 equivalents of Mg^2+^ (blue), 15 eq Mg^2+^ + 15 eq Ca^2+^ (red), and 15 eq Ca^2+^ (green). **(D–F)** Variation of ^1^H-^15^N HSQC peak intensities of WT CIB2 as a function of Ca^2+^ concentration. The peak intensities were normalized with respect to the maximum value. The continuous lines represent the data fitted against equations as indicated in section Materials and Methods. The plots refer to the amide peaks of residues E64 **(D)**, G162 **(E)**, and N121 **(F)**. All the spectra were recorded at 600 MHz and 25°C. All samples were at protein concentration of 320 μM in 20 mM Hepes, 100 mM KCl, 1 mM DTT, pH 7.5.

In line with previously reported data (Huang et al., 2012), the ^1^H-^15^N HSQC spectrum changed dramatically upon addition of either Ca^2+^ or Mg^2+^ ions; the NMR signals became more disperse and sharp and new downfield peaks appeared, indicative of metal binding (Figures 5A–C).

In order to investigate at a deeper level the structural mechanisms associated with Ca^2+^ binding, NMR titration experiments of Ca^2+^ into ^15^N-WT CIB2 have been performed collecting a series of ^1^H-^15^N HSQC spectra. Notably, the variation of the intensity of the peaks belonging to residues E64, which belongs to the kinked H3b helix in the non-functional EF1 motif and N121 of EF3 loop appeared to be correlated (Figures 5D,F), thus indicating that these amino acid residues belong to the same allosteric network. The NMR titration data were fitted assuming a simple one-site binding model which better describes a hyperbolic shape of the curve and *K*_*d*_ values of 0.55 ± 0.13 and 0.48 ± 0.15 mM were obtained for E64 and N121, respectively.

Moreover, we were able to follow the peculiar behavior of the G162 belonging to the EF4 loop, upon addition of increasing amount of Ca^2+^ (Figure 5E). Interestingly, the intensity variation upon addition of Ca^2+^ had a sigmoidal shape, indicative of positive cooperativity of the binding mechanism. From the fitting of the data a K_d_^app^ value of 2.22 ± 0.25 mM and a Hill coefficient h = 2.27 ± 0.38 were obtained. The data of the two sites with different affinities for the Ca^2+^ ion are consistent with the analysis of the ^1^H-^15^N HSQC spectra of the protein in the early steps of titration (data not shown). When only the peaks of E64 and N121 are visible, the protein already adopts a well-folded structure, further addition of Ca^2+^ promotes only small changes in the spectrum, thus confirming that the binding of Ca^2+^ into the first site triggers the structural rearrangement of WT CIB2.

NMR spectroscopy was also employed to investigate whether WT CIB2 had a preferential binding capability toward Ca^2+^ or Mg^2+^. To this aim we recorded a ^1^H-^15^N HSQC spectrum of ^15^N-WT CIB2 after addition of a solution containing equal concentration of the two cations (Figure 5C) and we analyzed the downfield peaks as indicators of the binding site occupancy. Interestingly, WT CIB2 displayed a preferential binding site for Mg^2+^ in the EF3 loop and for Ca^2+^ in the EF4 loop. This observation was confirmed by analyzing the ^1^H-^15^N HSQC spectra of the protein in the presence of different ratios of Ca^2+^ or Mg^2+^ (Figure S5). As expected, when Mg^2+^ was in excess we could observe the peak of N121 at the chemical shift of the Mg^2+^ bound form, and the peak of G162, although with low intensity, as a reporter of Ca^2+^ bound to the EF4 loop. Notably, when Ca^2+^ was in excess the protein still retained its capability to bind Mg^2+^ in EF3 and we could still observe the peak of N121, typical of the Mg^2+^ bound form, while EF4 became occupied by Ca^2+^.

### The affinity of WT and E64D CIB2 for Ca^2+^ is incompatible with a role as a physiological Ca^2+^ sensor

**Figure 6:**
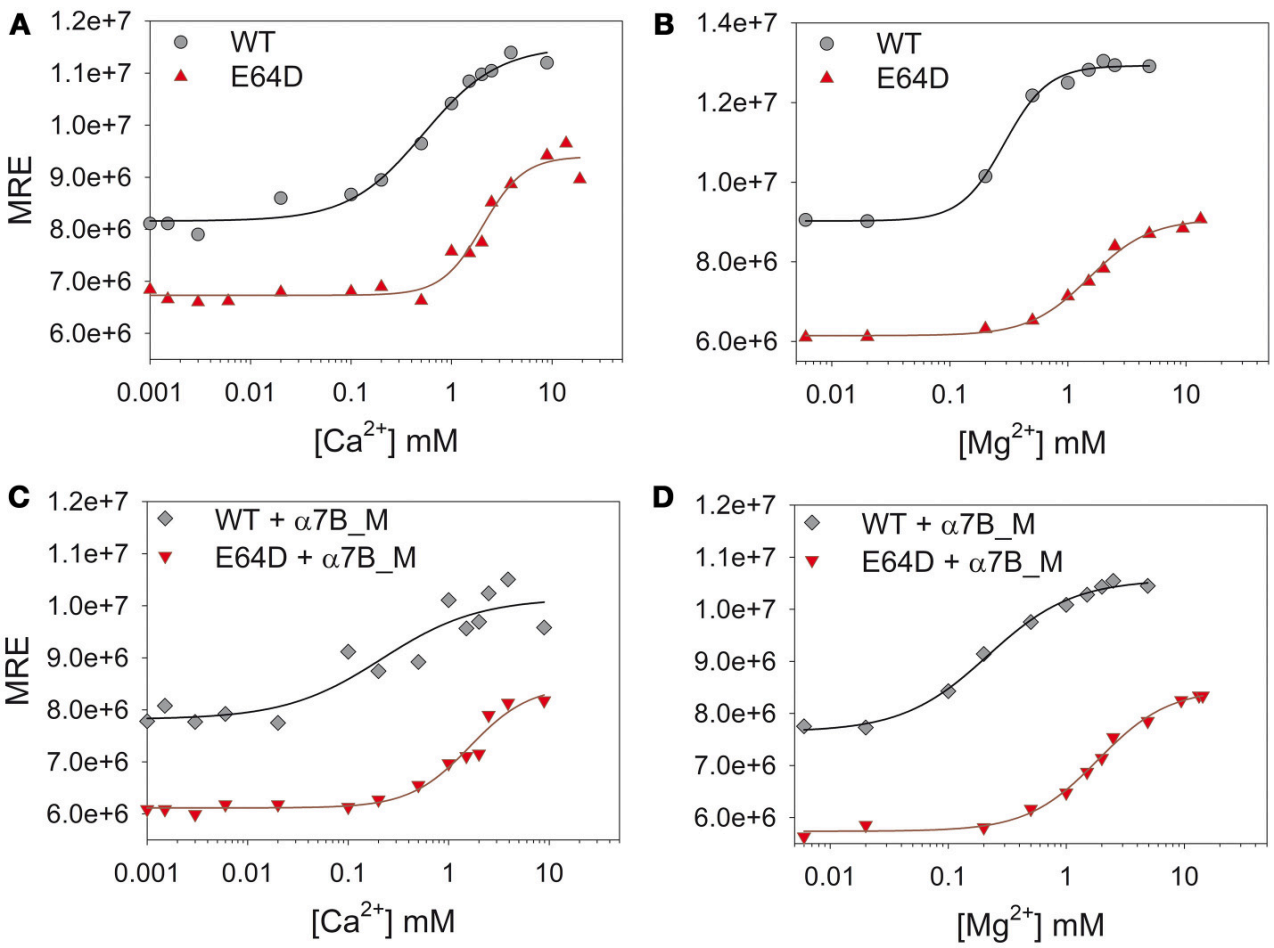
Ca^2+^ and Mg^2+^ titrations of CIB2 in the presence or in the absence of target peptide α7B_M. **(A,B)** WT (circles) and E64D (triangles) CIB2 were titrated with Ca^2+^ **(A)** and Mg^2+^ **(B)** starting from the apo-form (1 mM free EGTA). **(C,D)** Ca^2+^ **(C)** and Mg^2+^ **(D)** titrations of WT (diamonds) and E64D (downwards triangles) CIB2 in the presence of α7B_M peptide. Ions concentration ranged between 1 μM and 10 mM, the obtained data were fitted using the Hill 4 parameters function. Data were normalized on protein concentration (MRE). Titrations were performed at 25°C, each point represents the mean of 3 accumulations.

NMR experiments suggest that the affinity of WT CIB2 for Ca^2+^ is in the submillimolar range. We sought to quantify the affinity for Ca^2+^ and Mg^2+^ of both WT and E64D CIB2 in a comparative fashion in order to assess the potential role of CIB2 as a sensor protein under physiological and USH1J-related conditions. Titration experiments were performed by monitoring the CD signal at 222 nm, under very carefully determined pH and free cation (Ca^2+^ or Mg^2+^) conditions spanning over their known physiological range. Although the method does not allow attributing the macroscopic association constant to each individual EF-hand, it provides an estimate of the cation concentration at which the conformational change, starting from the apo-state, is half maximal (K_d_^app^). Therefore, it is a useful approach for comparisons of the two CIB2 variants over a physiological range of cation stimuli. Results are reported in Figure 6 and Table 1.

**Table 1:** Apparent affinity for Ca^2+^ and Mg^2+^ of WT and E64D CIB2 assessed by CD titrations.

	**Mg**^**2+**^		**Ca**^**2+**^	
	**K_d_^app^ (mM)**	**H_c_**	**K_d_^app^ (mM)**	**H_c_**
**WT CIB2**	0.29 ± 0.02	2.3 ± 0.3	0.5 ± 0.1	1.1 ± 0.3
**WT** + α**7B_M**	0.49 ± 0.03	1.4 ± 0.1	0.2 ± 0.2	0.9 ± 0.6
**E64D CIB2**	1.5 ± 0.1	1.7 ± 0.2	2.0 ± 0.2	2.2 ± 0.6
**E64D** + α**7B_M**	1.8 ± 0.2	1.4 ± 0.2	1.5 ± 0.3	1.4 ± 0.3

In line with the data from NMR titrations, the measured apparent affinity for Ca^2+^ of both WT (500 μM) and E64D (2 mM) CIB2 was extremely low, thus excluding a possible role of CIB2 as a Ca^2+^ sensor under physiological conditions (see the section Discussion for further details). An alternative well-established spectroscopic method based on the competition with the chromophoric chelator 5,5′Br_2_-BAPTA was applied for WT CIB2, but it failed to detect any individual macroscopic binding constant in the low μM range (results not shown), thus confirming the overall low affinity for Ca^2+^. Interestingly, WT CIB2 showed a relatively high affinity for Mg^2+^ (290 μM), compatible with a fully loaded state under physiological conditions. On the contrary, E64D CIB2 is likely incapable of detecting Mg^2+^ under physiological conditions due to the low affinity (K_d_^app^ = 1.5 mM).

All the titration curves could be fitted to a Hill sigmoid function (Figure 6), thus suggesting in some cases a cooperative effect of the cation binding on the structural transition. Interestingly, while Ca^2+^ binding to WT CIB2 was substantially non-cooperative (H_c_ = 1.1, Table 1), binding of Mg^2+^ showed evidence of positive cooperativity (H_c_ = 2.3, Table 1). As for E64D CIB2, data suggest positive cooperativity both in the case of Mg^2+^ (H_c_ = 1.7, Table 1) and Ca^2+^ binding (H_c_ = 2.2, Table 1).

The fact that we detected for E64D CIB2 a significantly low affinity for both Ca^2+^ and Mg^2+^ (Table 1) made us wonder if higher concentration of cations could trigger a WT-like conformation. Near UV-CD spectra were thus recorded following sequential additions of increasing Ca^2+^ or Mg^2+^ (Figure S6). In line with the results from far UV-CD spectroscopy and titration experiments, our data show that at a Ca^2+^ concentration up to 5 mM E64D CIB2 did not switch to a WT-like three-dimensional conformation; however, at 10 mM Ca^2+^ the near UV spectra became similar (compare Figure S6a with Figure 3B). Interestingly, the same finding did not apply to Mg^2+^. Even at 10 mM Mg^2+^ the near UV CD spectrum did not reach the shape and the intensity observed for the WT case (compare Figure S6b with Figure 3E). Therefore, our data are overall consistent with the inability of E64D CIB2 to sense Mg^2+^ under physiological conditions, and even under conditions that exceed the intracellular levels.

### α7B integrin is a specific target of both WT and E64D CIB2

**Figure 7:**
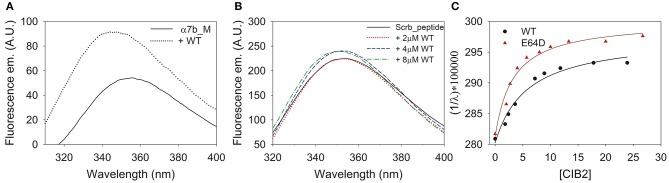
Interaction between CIB2 variants and α7B_M peptide monitored by fluorescence spectroscopy. **(A)** Trp fluorescence emission spectra of 4 μM α7B_M peptide alone (solid line) and incubated with of 8 μM CIB2 WT) (dotted black line) in the presence of 1 mM Mg^2+^ and 1 mM ${\text{Ca}}_{.}^{2+}$ **(B)** Trp fluorescence emission spectra in 1 mM Mg^2+^ and 1 mM Ca^2+^of 4 μM Scrb peptide alone (solid black line) and in the presence of 2 μM CIB2 WT (dotted red line), 4 μM CIB2 WT (short dashed blue line) and 8 μM CIB2 WT (dash dotted green line). **(C)** Fluorescence titration of 4 μM α7B_M peptide with WT (black circles) and E64D (red triangles) CIB2 in the presence of 1 mM Mg^2+^ and 1 mM Ca^2+^.

We asked whether E64D CIB2 was still capable of interacting with specific targets of WT CIB2. Based on the results of previous work (Huang et al., 2012), we focused on a peptide (α7B_M) covering the membrane-proximal CIB2-specific sequence of recognition of the integrin α7B cytosolic domain. As a negative control, we generated a scrambled peptide (Scrb) by shuffling the α7B_M sequence, thus conserving general physicochemical properties such as net charge and hydrophilicity while losing biological specificity. Both peptides have a single Trp residue, which allowed us to exploit their fluorescence for studying the interaction with both WT and E64D CIB2, which lack Trp residues (see section Materials and Methods). Results from fluorescence experiments are reported in Figure 7.

The interaction between WT CIB2 and the α7B_M peptide was apparent, as assessed from the 1.7-fold increase in the maximal fluorescence emission and the 12 nm blues shift (Figure 7A) indicative of an augmented hydrophobicity of the peptide Trp residue upon interaction with the protein. On the contrary, no significant change in either the fluorescence emission intensity and the relative maximum wavelength was observed when the experiments were performed with the Scrb peptide, even when the concentration of WT CIB2 was brought up to 8 μM, thus indicating the lack of specific binding (Figure 7B). Similar results were obtained for the E64D CIB2 variant (results not shown).

Titration experiments were performed to estimate the stoichiometry of interaction between WT/E64D CIB2 and α7B_M peptide and to assess the apparent affinity. Results are reported in Figure 7C. The curves showed that both WT and E64D CIB2 interact with the target peptide with a 2:1 stoichiometry, that is, a CIB2 dimer binds a single peptide. The estimated affinities are similar (K_d_^app^ = 4.99 ± 1.01 μM for WT CIB2; K_d_^app^ = 3.1 ± 0.2 μM for E64D CIB2; mean ± s.d. of 4 and 3 repetitions, respectively), therefore the USH1J-related variant is still capable of binding the α7B_M target peptide, with even higher affinity compared to the WT case.

In order to assess if the binding of the target peptide could influence the sensing of Ca^2+^ or Mg^2+^ and the protein conformation, far UV CD spectra were recorded and titration experiments performed in the same conditions as with the protein alone. Figure S7 clearly shows that the peptide does not possess any secondary structure and that its incubation with WT CIB2 led to the same spectral properties observed in response to additions of Ca^2+^ for the protein alone (compare with Figure 3C). Therefore, we conclude that the interaction with the α7B_M peptide does not induce any appreciable structural change in WT CIB2. Moreover, the interaction with the α7B_M peptide had a relatively small effect on the Ca^2+^ or Mg^2+^ sensing abilities of CIB2. While a 1.6-fold increase in the K_d_^app^ was observed for Mg^2+^ binding to WT CIB2 (Table 1 and Figure 6), a slightly increased affinity for Ca^2+^ was detected, although the K_d_^app^ was still quite high for physiological relevance (0.2 mM, Table 1). Minor differences were observed in the variation of K_d_^app^ in the presence of α7B_M peptide for E64D CIB2 (1.2-fold increase for Mg^2+^ and 1.2-fold decrease for Ca^2+^; Figure 6 and Table 1).

## Discussion

The ubiquitous expression of CIB2 in various tissues suggests that it may exert yet unknown biological functions in a broad range of biochemical processes. Besides being involved in hearing physiology and pathology (Riazuddin et al., 2012; Jan, 2013; Patel et al., 2015; Seco et al., 2016; Wang et al., 2017), CIB2 has been indeed found to play a role in congenital muscular dystrophy type 1A (Häger et al., 2008), in the N-methyl-D-aspartate receptor-mediated Ca^2+^ signaling in cultured hippocampal neurons (Blazejczyk et al., 2009), in the promotion of HIV-viral infection (Godinho-Santos et al., 2016), and very recently it was found to act as a negative regulator of sphingosine kinase 1-mediated oncogenic signaling in ovarian cancer (Zhu et al., 2017). Available mechanistic studies focusing on the Ca^2+^ and Mg^2+^ sensing properties of CIB2 are just a few, and a comprehensive characterization of the protein in comparison to its disease-associate variants was missing. Indeed, so far much of the molecular interpretation of the processes in which CIB2 is involved has been based on the significantly better explored structure-function properties of the homologous protein CIB1 (Leisner et al., 2016), although the relatively low sequence identity and similarity call for particular caution when inferring common functions for the two proteins.

In this work, we present a thorough characterization of two variants of human CIB2, namely the WT form and the E64D mutant associated with USH1J. It should be reported that a recent study disqualified CIB2 as a USH1J-related gene, however the E64D variant was found to be associated with autosomal recessive non-syndromic hearing loss (Booth et al., 2018). Our biochemical and biophysical study highlights a number of clear structural and functional differences with CIB1, which may thus pose the molecular basis for understanding the malfunctioning of CIB2 in USH1J and possibly other genetic diseases causing hearing loss.

While the general topology of CIB2 is similar to that of CIB1 (Figure 1), a first clear difference between CIB1 and CIB2 resides in their oligomeric states. While analytical SEC experiments performed with CIB1 detected a monomeric protein independently on the presence of Ca^2+^ and target peptide (Gentry et al., 2005), our SEC data, electrophoresis experiments under non-denaturing conditions (Figure S2 and Table S1) and DLS experiments (Figure 2) all converge to CIB2 forming non-covalent dimers both in the apo and in Ca^2+^/Mg^2+^-bound conditions. The oligomeric state of CIB2 is particularly relevant for its interaction with biological targets. Although we cannot exclude different situations with different targets, fluorescence titration experiments (Figure 7) point to a 1:1 stoichiometry for a CIB2 dimer:α7B_M peptide complex, at odds with the results observed for CIB1:αIIb peptide complex, where the 1:1 stoichiometry involved a monomeric protein (Gentry et al., 2005).

The stability of the oligomeric state of WT CIB2 was found to be significantly affected by the presence of metal cations and by the presence of the E64D point mutation. DLS spectroscopy highlighted how, in order to achieve a substantially monodisperse protein solution, the saturation with Ca^2+^ or Mg^2+^ was necessary, as the apo-form was observed to dynamically fluctuate between oligomers of different size (Figure 2 and Figure S3). Surprisingly, the USH1J-associated E64D mutation, that does not change the physicochemical properties of the substituted amino acid, still leads to a dimeric protein (Figure S2), which is however more prone to form heterogeneous aggregates over time independent of the presence of Ca^2+^ or Mg^2+^ (Figure 2 and Figure S3).

Important differences between WT and E64D CIB2 were found in their cation-dependent folding state. A general agreement between ^1^H NMR and near and far UV-CD spectra was obtained for both protein variants (Figure 3). Indeed, WT CIB2 was found to respond to both Ca^2+^ and Mg^2+^ by adopting a similar secondary (Figure 3C) and tertiary structure (Figures 3A,B), at odds with the E64D variant, for which 1 mM Mg^2+^ was not enough to trigger any detectable switch (Figures 3D–F). Further addition of 1 mM Ca^2+^ led to a detectable increase in E64D CIB2's tertiary structure (Figures 3D,E) although the change was significantly lower compared to that observed for the WT. Besides showing a lower structural responsiveness to Ca^2+^ and Mg^2+^ compared to the WT, E64D CIB2 was found to have a significantly lower thermal stability under all the tested conditions (Figure 4 and Table S2), and its apo-form apparently maintains a more hydrophobic surface that persists upon exposition to Ca^2+^ and Mg^2+^ (Figure S4). This could also explain while no transition was observed for apo E64D CIB2 upon thermal denaturation in the 4–70°C range (Figure 4B), this form being particularly unstable and unstructured.

2D HSQC NMR experiments shed light on the mechanisms related to Ca^2+^ and Mg^2+^ binding to WT CIB2 (Figure 5). While confirming the molten globule conformation of the apo form, NMR highlighted that the Ca^2+^- and Mg^2+^-bound states of WT CIB2 have a rather similar three-dimensional structure. The analysis of the downfield regions permits the distinction of specific Ca^2+^- or Mg^2+^-related fingerprints in the observed pattern. In particular, by performing Ca^2+^ titrations we observed that the intensity of the peak attributed to N121, which is located in the sixth position of the EF3 metal binding loop (Figure 1) shows a very similar trend compared to that of E64, the residue substituted by Asp in USH1J, which is located in the N-terminal domain, far from the metal binding loops (Figure 1). This surprising finding suggests that an inter-domain allosteric communication occurs between the EF3 binding loop and E64, which according to the homology model based on the structure of CIB1, forms an electrostatic interaction with R33 (Figure 1) and is therefore likely contributing to the stability of the EF1 subdomain. The titration patterns observed by NMR (Figures 5D–F) further confirm that EF3 is the first EF-hand to be occupied by Ca^2+^, followed by EF4, whose structural probe is the G162 residue in the sixth position of the loop (Figure 5E). Our data support a model, in which under physiological conditions EF3 is never occupied by Ca^2+^ but is instead always Mg^2+^-bound (Figure S5). Ca^2+^ will however bind to the EF4 loop under conditions of particularly high Ca^2+^ concentration. Moreover, no replacement of Mg^2+^ was observed in EF3 following additions of equal amounts of Ca^2+^ into Mg^2+^-bound WT CIB2 (Figure 5C).

These findings appear particularly relevant for their physiological implications when considered together with the estimated affinities for Ca^2+^ and Mg^2+^ of WT and E64D CIB2 (Figure 6 and Table 1). The intracellular concentration of free Ca^2+^ oscillates in the 0.1–10 μM range (Berridge et al., 1998, 2000) and it is even lower in the outer segments of photoreceptor cells, where a fine regulation of the phototransduction cascade by Ca^2+^ and cGMP operates (Koch and Dell'Orco, 2013, 2015). The level of free Mg^2+^ in most cells, however, is relatively constant and ranges in the 0.5–1 mM interval (Romani and Scarpa, 1992, 2000). While a 290 μM apparent affinity for Mg^2+^ (Table 1) is consistent with the binding of Mg^2+^ to WT CIB2 under physiological conditions, the affinity measured for the E64D variant (1.5 mM) is too low for ensuring sensing capabilities under normal conditions. Moreover, neither WT nor E64D CIB2 could possibly work as Ca^2+^ sensors with the apparent affinities detected in our study (500 μM and 2 mM, respectively). It should be noticed that other authors (Blazejczyk et al., 2009) previously determined a much higher affinity for Ca^2+^ for GST-fused CIB2 by using a TNS fluorescence assay (apparent *K*_*d*_ = 0.l4 μM). Such a high affinity is in contrast with our data based on three different experimental approaches, namely NMR and CD spectroscopic titrations and competition experiments with the 5,5′Br_2_-BAPTA chromophoric chelator. This latter approach excluded apparent *K*_*d*_ values below 6 μM (results not shown) and was instead able to detect binding of Ca^2+^ to CIB1 (Yamniuk et al., 2008). We don't have an explanation for such discrepancy, except for pointing out that all our experiments were performed with unlabeled and untagged human proteins, while those in Blazejczyk et al. used a TNS-labeled rat CIB2 fused with GST, which might introduce artifacts when probing the protein sensing capabilities.

The lower affinity for Ca^2+^ is one of the elements distinguishing CIB2 from CIB1, which binds Ca^2+^ with high affinity in EF4 (*K*_*d*_ = 0.5 μM) and with lower affinity in EF3 (*K*_*d*_ = 1.9 μM); binding of Mg^2+^ is instead limited to EF3 (*K*_*d*_ = 120 μM) (Yamniuk et al., 2004, 2007). A closer look at the sequence alignment of the EF3 and EF4 Ca^2+^-binding motifs (Figure 1) explains, at least in part, such difference. The high affinity of the EF4 loop for Ca^2+^ in CIB1 can be attributed to the optimal pentagonal bipyramid geometry of the Ca^2+^-coordinating oxygens, also due to the presence of an Asn residue (N169) in the -X position and especially to a Glu residue in the -Z position (E172). This latter constitutes a bidentate ligand providing the highly conserved coordination via the two γ-carboxyl groups to the Ca^2+^ ion (Gagné et al., 1997; Grabarek, 2011). In CIB2, positions -X and -Z are occupied respectively by G165, lacking contributions from the side chain, and D168, which does not possibly act as a bidentate ligand (Figure 1). Moreover, the position occupied by the side chain of D159 in the structural model of CIB2 does not seem optimal for contributing to Ca^2+^ coordination even after energy-minimization, at odds with that of the aligned residue D163 in CIB1 (Figure 1). Overall, the geometry of the Ca^2+^-coordinating oxygens in the EF4 loop of CIB2 is thus likely distorted with respect to the canonical one, hence leading to a low affinity for Ca^2+^. Differences in the sequence of the EF3 loop also distinguish CIB2 from CIB1, which may explain the lower affinity for Ca^2+^ shown by the first protein. Position Y in CIB1 is occupied by the negatively charged D118, which is substituted by N118 in CIB2, moreover the -X position, again occupied by an Asn (N124) in CIB1 is occupied by a Cys (C124) in CIB2. Interestingly, CIB1 like CIB2 does not have a Glu at position -Z, but has an Asp instead (D127), thus explaining the lower affinity for Ca^2+^-compared to EF4.

The fact that all four CIBs have an Asp instead of a Glu residue at position -Z (Figure 1) suggests that EF3 can serve as a Mg^2+^-binding specific motif. Previous mutagenesis studies showed that the replacement of the Asp residue in the 12th position (-Z) of an EF-hand metal binding loop with a Glu increases the affinity for Ca^2+^ and abolishes binding of Mg^2+^, rendering the site calcium-specific, probably due to the reduced ability of side chains to change conformation (da Silva et al., 1995). We are therefore tempted to generalize that EF3 is the Mg^2+^-specific binding motif among the CIB family, while Ca^2+^ might bind to EF4 under physiological conditions, although this is clearly not the case for CIB2. The conformational switch from a molten globule to a well-defined tertiary structure is likely governed by the acidic residue in the 12th position (-Z) of an EF-hand (Gifford et al., 2007), therefore Mg^2+^ seems to be the initiator of the functional switch among the CIB family.

We have also studied the potential effect of a CIB2-specific target on the protein metal cation-sensing ability. Although our investigation has been limited to one of the many possible binding targets of CIB2, namely a peptide covering the membrane-proximal sequence of the integrin α7B cytosolic domain, our data show that the interaction with the target doubles the apparent affinity of CIB2 for Ca^2+^ (Table 1), however the detected K_d_^app^ is still incompatible with a physiological capability of Ca^2+^ sensing. We cannot exclude, however, that in specific cell compartments and/or under specific conditions related to disease and/or cell death, the increased intracellular Ca^2+^ and the concomitant presence of a specific target would render CIB2 capable of Ca^2+^ sensing, therefore providing specific functions. This is however not possibly the case of E64D CIB2, which showed a mM affinity for Ca^2+^ under all the tested conditions, including the presence of the target peptide (Table 1 and Figure 6).

Both WT and E64D CIB2 were shown to bind specifically the α7B_M target peptide with a low μM affinity comparable to that shown for CIB1-αIIb interaction (*K*_*d*_ = 1.41–1.02 μM, in the presence of Ca^2+^ and Mg^2+^, respectively) (Shock et al., 1999; Yamniuk and Vogel, 2005). Future studies will be necessary to further elucidate the binding thermodynamics of CIB2 to its putative targets, including those belonging to the Usher interactome. A broader set of conditions including different cation concentrations and models that account for the dimeric nature of CIB2 shall be specifically tested. Nevertheless, our data seem sufficient to exclude that the principal dysfunction of the USH1J-associated E64D CIB2 be related to the lack of recognition of specific targets. Instead, our data point clearly to the incapability of this CIB2 mutant to switch to its native, Mg^2+^-bound conformation (Figures 3, 5, 6). E64D CIB2 was indeed observed to maintain, under physiological levels of Mg^2+^, a partially unfolded conformation that makes it significantly less stable and prone to aggregation compared to the WT (Figure 2 and Figure S3).

The switch that allows WT CIB2 to acquire a functional conformation at physiological Mg^2+^ appears to be finely regulated by an allosteric, long-range communication connecting EF1 with EF3. Our data are inconsistent with mutations in CIB2 disrupting auditory hair cell calcium homeostasis (Jan, 2013) as with such a low affinity not even the WT protein is expected to be involved in Ca^2+^ sensing under physiological conditions. Instead, we propose that the inability to bind Mg^2+^ of E64D CIB2 prevents the allosteric regulation that makes the protein switch to the native conformation required for its normal function.

## Author contributions

RV, GD, MD, and DD planned the experiments and analyzed the results. RV, GD, and MD performed the experiments. DD wrote the manuscript with contributions from all the authors.

### Conflict of interest statement

The authors declare that the research was conducted in the absence of any commercial or financial relationships that could be construed as a potential conflict of interest.
