# Supplementary material for: Preferential Binding of Mg2+ Over Ca2+ to CIB2 Triggers an Allosteric Switch Impaired in Usher Syndrome Type 1J
Source: Front Mol Neurosci. 2018 Aug 17;11:274. doi: 10.3389/fnmol.2018.00274 (PMC6107761; doi:10.3389/fnmol.2018.00274)
Supplement: Supplementary file 1 [file Data_Sheet_1.PDF]

*Supplementary Material*

# Preferential binding of $Mg^{2+}$ over $Ca^{2+}$ to CIB2 triggers an allosteric switch impaired in Usher Syndrome type 1J

**Rosario Vallone, Giuditta Dal Cortivo, Mariapina D'Onofrio and Daniele Dell'Orco***

*** Correspondence:** daniele.dellorco@univr.it

**Figure S1**

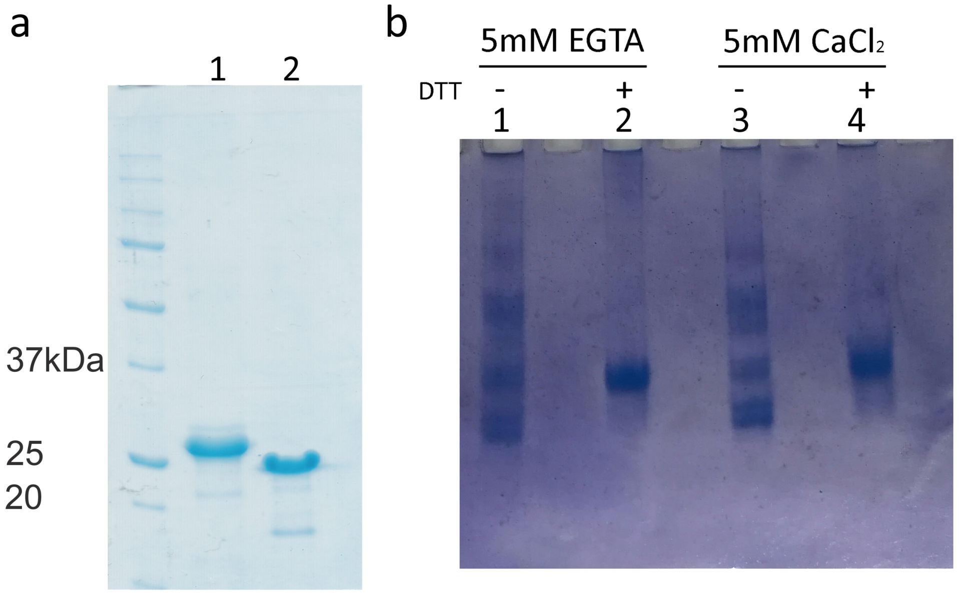


**Figure S1**: SDS and Native-PAGE of CIB2. **a**) SDS-PAGE of 20μM CIB2 before (lane 1) and after (lane 2) TEV-cleavage of the His-tag in the presence of 1mM DTT. **b**) Continuous native-PAGE (12% acrylamide/bis- acrylamide) of 14μM CIB2 in the presence of 5mM EGTA (lanes 1-2) or 5mM $Ca^{2+}$ (lanes 3-4). 1mM DTT was added in lanes 2 and 4.

**Figure S2**

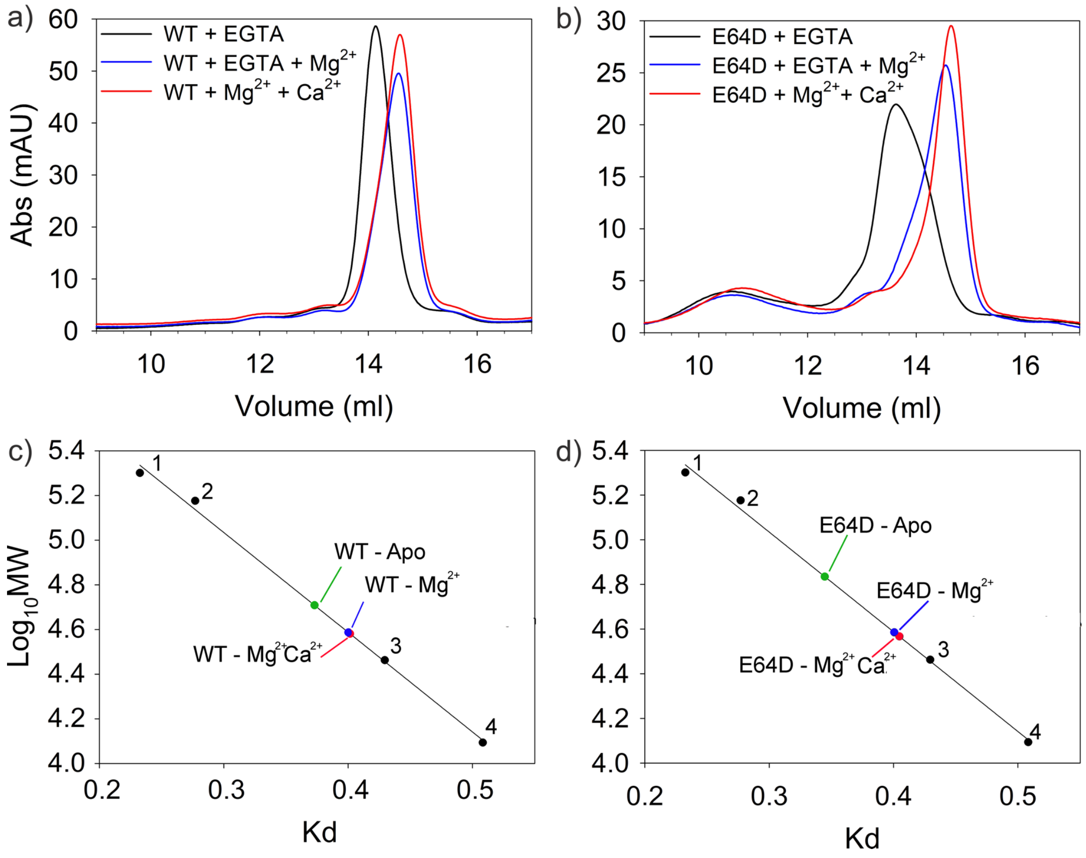


**Figure S2:** SEC analysis. Gel filtration elution profiles under reducing conditions of : **a**) WT CIB2 and **b**) E64D CIB2 in the presence of 3 mM EGTA (black line), 3 mM $Mg^{2+}$ + 2 mM EGTA (blue line) and 3 mM $Mg^{2+}$ + 2 mM $Ca^{2+}$ (red line). **c-d**) Determination of MW for apo-, $Mg^{2+}$ and $Ca^{2+}$ -bound WT and E64D CIB2. Calibration curve prepared by plotting the $Log_{10}$ MW value for each standard protein versus its corresponding $K_d$ value. 1. β-amylase; 2. Alcohol Dehydrogenase; 3. Carbonic anhydrase; 4. Cytochrome c. Chromatograms in a) and b) were used to determine the MW for apo-, $Mg^{2+}$ and $Ca^{2+}$ -bound WT CIB2 and E64D CIB2, respectively (Table S1).

**Figure S3**

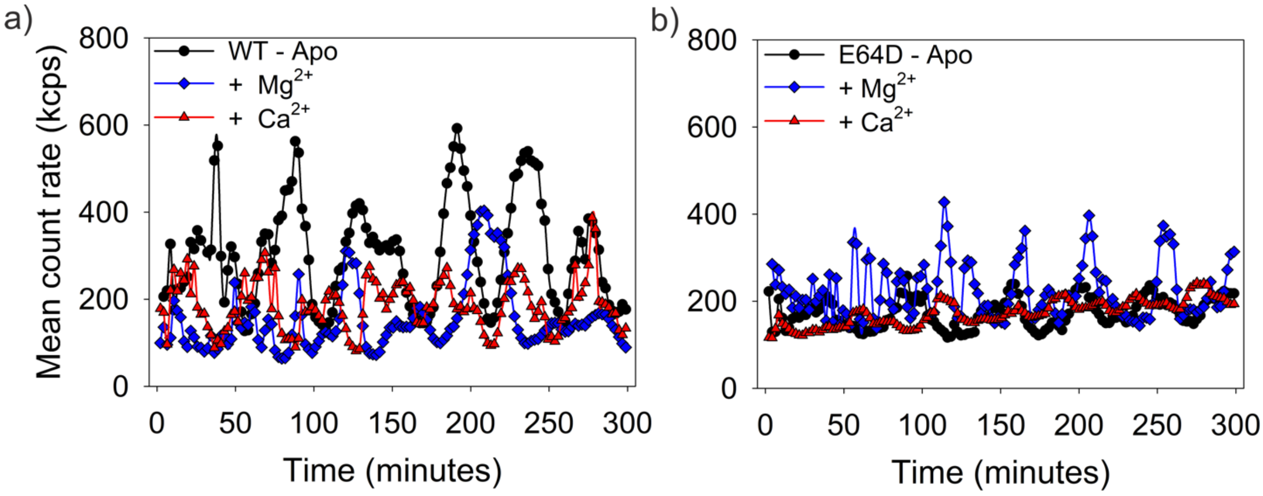


**Figure S3:** Time-resolved dynamic light scattering. Mean count rate time-course of **a)** WT and **b)** E64D CIB2 in the presence of 3 mM EGTA (black line), 3 mM $Mg^{2+}$ + 2 mM EGTA (blue line) and 3 mM $Mg^{2+}$ + 2 mM $Ca^{2+}$ (red line).

**Figure S4**

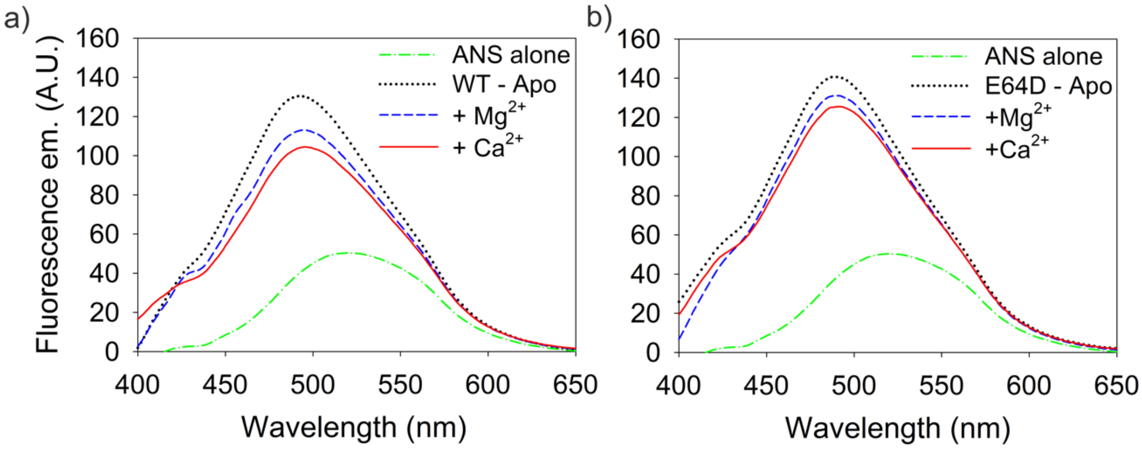


**Figure S4: ANS fluorescence spectroscopy.** ANS fluorescence spectra of: **a)** WT CIB2 and **b)** E64D CIB2 in the presence of 0.5 mM EDTA (dotted black line), 1 mM $Mg^{2+}$ (short dashed blue line) and 1 mM $Mg^{2+}$ + 1 mM $Ca^{2+}$ (solid red line). The fluorescence spectrum of ANS alone is also shown (dash dotted green line).

## Figure S5

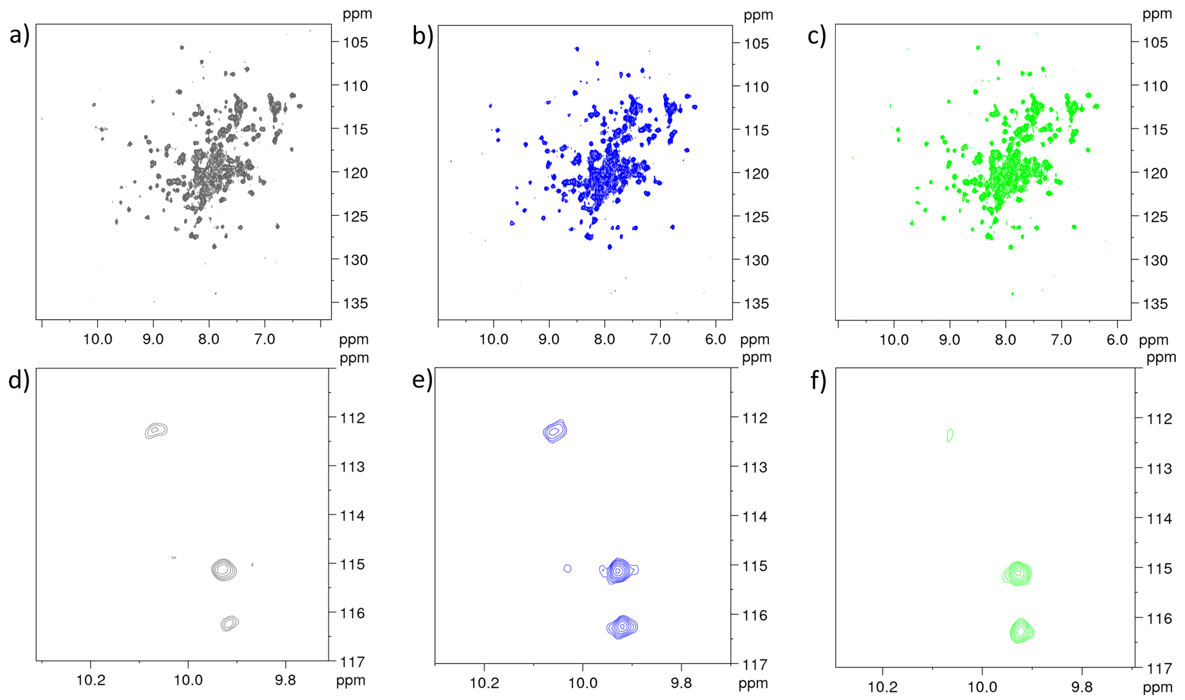


**Figure S5: 2D NMR spectra.** Two-dimensional $^{1}H$-$^{15}N$ HSQC NMR spectra of $^{15}N$-WT CIB2 with addition of 3 mM $Mg^{2+}$ + 7 mM $Ca^{2+}$ (panels **a**, **d**), 5 mM $Mg^{2+}$ + 5 mM $Ca^{2+}$ (panels **b**, **e**), 7 mM $Mg^{2+}$ + 3 mM $Ca^{2+}$ (panels **c**, **f**). The complexes were obtained by adding to the protein different solutions containing both $Mg^{2+}$ and $Ca^{2+}$ ions yielding the same overall ionic strength. The spectra were recorded at 600 MHz and 25°C, the samples were at protein concentration of 150 µM in 20 mM Hepes, 100 mM KCl, 1 mM DTT, pH 7.5 and 7% $D_2O$.

## Figure S6

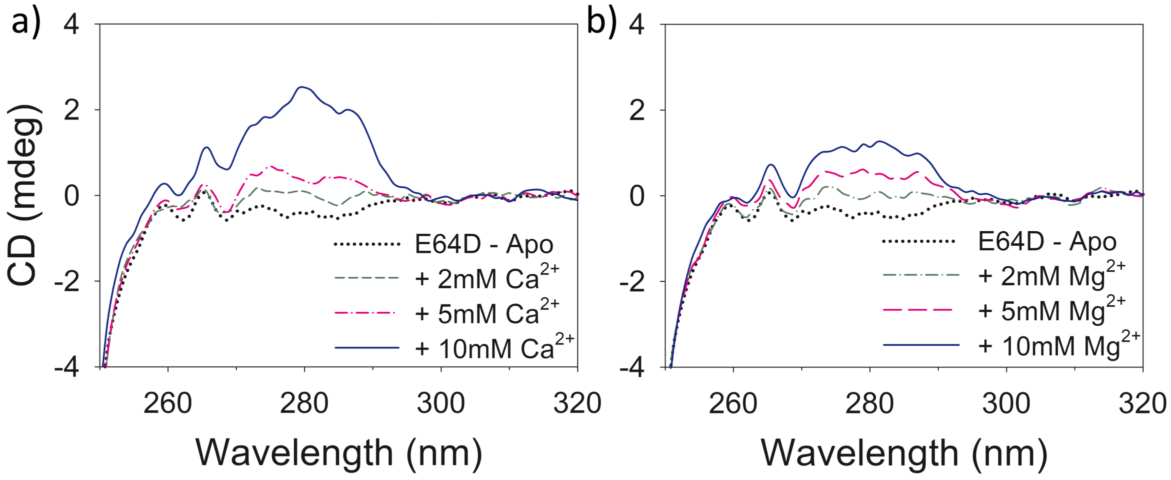


**Figure S6:** Near UV CD spectra of E64D CIB2 after sequential additions of increasing concentrations of: **a**) $Ca^{2+}$ and: **b**) $Mg^{2+}$ in the 2-10 mM range. The experimental conditions were the same as those of ***Figure 3e***.

**Figure S7**

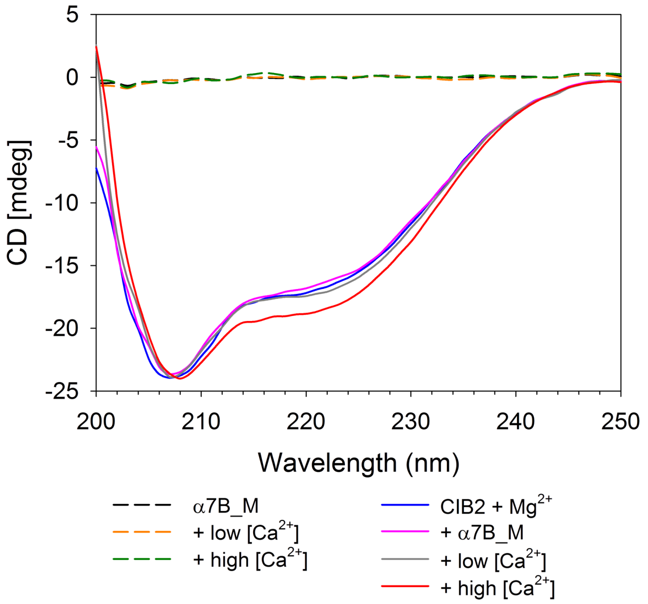


**Figure S7: Effect of the α7B_M peptide on the secondary structure of WT CIB2.** 12µM $Mg^{2+}$ bound-WT CIB2 was incubated at 37°C in the presence of 6µM α7B_M peptide (ratio 2:1). Spectra were collected in the presence of low (100µM, grey line) and high (1mM, red line) $Ca^{2+}$ concentration. Spectra obtained for the α7B_M peptide in the absence of CIB2 are also shown (dashed lines).

**Table S1: Molecular weight of WT and E64D CIB2 estimated by Analytical SEC and Ferguson Plots**

| | $MW^{SEC}$ (KDa) | $MW^{FP}$ (KDa) |
|---|---|---|
| **WT -apo** | 51.1 | 51 |
| **+ $Mg^{2+}$** | 38.6 | 52 |
| **+$Mg^{2+}$+ $Ca^{2+}$** | 38.1 | 53 |
| **E64D -apo** | 68.4 | 53 |
| **+ $Mg^{2+}$** | 38.5 | 53 |
| **+$Mg^{2+}$ + $Ca^{2+}$** | 36.9 | 53 |

**Table S2: Analysis of ANS fluorescence and CD thermal denaturation data for WT and E64D CIB2**

| | blue shift (nm)[1] | $F^{max}/F^{ref}$ | $T_m$ (°C) | $H_c$ |
|---|---|---|---|---|
| **WT -apo** | 27 | 2.6 | 35.1 | 7.5 |
| **+ $Mg^{2+}$** | 25 | 2.2 | 45.7 | 11 |
| **+ $Ca^{2+}$** | 25 | 2.1 | 43.4 | 12.5 |
| **+$Mg^{2+}$/$Ca^{2+}$** | - | - | 45.9 | 11.2 |
| **E64D –apo** | 31 | 2.8 | - | - |
| **+ $Mg^{2+}$** | 30 | 2.6 | 34.2 | 9.5 |
| **+ $Ca^{2+}$** | 29 | 2.5 | 34.5 | 8.6 |
| **+$Mg^{2+}$/$Ca^{2+}$** | - | - | 36.7 | 8.9 |

[1] Blue shift refers to the observed shift of the wavelength of maximum emission of the protein + ANS mixture upon excitation at 380 nm as referred to that of ANS alone.

[2] $F^{max}/F^{ref}$ refers to the ratio between maximal fluorescence emission of the protein + ANS mixture and that of ANS alone.

[3] $T_m$ and $H_c$ represent, respectively, the melting temperature and the Hill coefficient obtained by data fitting in thermal denaturation profiles.
